# Population genomic analyses of RAD sequences resolves the phylogenetic relationship of the lichen-forming fungal species *Usneaantarctica* and *Usneaaurantiacoatra*

**DOI:** 10.3897/mycokeys.43.29093

**Published:** 2018-12-12

**Authors:** Felix Grewe, Elisa Lagostina, Huini Wu, Christian Printzen, H. Thorsten Lumbsch

**Affiliations:** 1 Integrative Research Center, Science and Education, Field Museum of Natural History, 1400 S Lake Shore Drive, Chicago, IL 60605, USA; 2 Department of Botany and Molecular Evolution, Senckenberg Research Institute and Natural History Museum Frankfurt, Senckenberganlage 25, 60325 Frankfurt/Main, Germany; 3 Department of Cell and Molecular Physiology, Stritch School of Medicine, Loyola University Chicago, 2160 S First Avenue, Maywood, IL 60153, USA

**Keywords:** Antarctica, Ascomycota, lichens, Parmeliaceae, phylogeny, RADseq

## Abstract

Neuropogonoid species in the lichen-forming fungal genus *Usnea* exhibit great morphological variation that can be misleading for delimitation of species. We specifically focused on the species delimitation of two closely-related, predominantly Antarctic species differing in the reproductive mode and representing a so-called species pair: the asexual *U.antarctica* and the sexual *U.aurantiacoatra*. Previous studies have revealed contradicting results. While multi-locus studies based on DNA sequence data provided evidence that these two taxa might be conspecific, microsatellite data suggested they represent distinct lineages. By using RADseq, we generated thousands of homologous markers to build a robust phylogeny of the two species. Furthermore, we successfully implemented these data in fine-scale population genomic analyses such as DAPC and fineRADstructure. Both *Usnea* species are readily delimited in phylogenetic inferences and, therefore, the hypothesis that both species are conspecific was rejected. Population genomic analyses also strongly confirmed separated genomes and, additionally, showed different levels of co-ancestry and substructure within each species. Lower co-ancestry in the asexual *U.antarctica* than in the sexual *U.aurantiacoatra* may be derived from a wider distributional range of the former species. Our results demonstrate the utility of this RADseq method in tracing population dynamics of lichens in future analyses.

## Introduction

Over the last decades, the use of DNA sequence data to delimit species and reconstruct phylogenetic relationships has become standard (Barraclough and Nee 2001; de Queiroz 2007; Holder and Lewis 2003; Huelsenbeck et al. 2001; Taylor et al. 2000; Wiens and Penkrot 2002). In groups with high morphological plasticity and homoplasy in phenotypical data sets, such as fungi, molecular data have dramatically changed our understanding of evolution and coinciding taxonomic interpretations (Hibbett et al. 2007; James et al. 2006; Lutzoni et al. 2004; McLaughlin et al. 2009; Robbertse et al. 2006; Schoch et al. 2009; Spatafora et al. 2017; Spatafora and Robbertse 2010; Stajich et al. 2009).

The general lineage species concept (de Queiroz 2007) allows researchers to use different empirical data to test the hypothesis of lineage separation, including phenotypical characters and molecular data. The latter dataset often provides strong evidence if analysed within a rigorous statistical framework (Rannala 2015). With regards to species delimitation, numerous studies of lichen-forming fungi detected distinct lineages lacking obvious distinguishing phenotypical characters, the so-called cryptic species (Bickford et al. 2007; Crespo and Lumbsch 2010; Crespo and Pérez-Ortega 2009; Lumbsch and Leavitt 2011). However, some studies also demonstrated that morphologically distinct populations could not be separated using single- or multi-locus genetic data. These results have been interpreted either as an indication of recent diversification and incomplete lineage sorting (Leavitt et al. 2016a; Zhao et al. 2017) or that the phenotypes represented populations of the same species (Articus et al. 2002; Buschbom and Mueller 2006; Kotelko and Piercey-Normore 2010; Lohtander et al. 1998; Myllys et al. 2001; Velmala et al. 2009). The latter result was often found in so-called species pairs. These are lichens that differ in forming either ascomata and reproducing sexually or forming asexual diaspores (soredia), which propagate the fungal and photosynthetic partner simultaneously (Mattsson and Lumbsch 1989; Poelt 1970; Tehler 1982). Otherwise, these species are morphologically identical, but were traditionally regarded as distinct species due to their different reproductive modes (Poelt 1972). The *Parmotremaperforatum* group was used as a model system of species delimitation based on the reproductive mode and secondary metabolites (Culberson and Culberson 1973). However, a recent study suggests that the phylogenetic relationships between sexual and asexual populations might be more complex (Widhelm et al. 2016).

We here focus on a complex of two morphologically similar species that differ in their reproductive mode and are considered a species pair in the genus *Usnea*: *U.aurantiacoatra* and *U.antarctica*, the latter reproducing by asexual soredia (Walker 1985). Within the genus, there is group of species predominantly occurring in Antarctica and adjacent cool-temperate to polar regions with a thallus that consists of yellow (containing usnic acid) and blackish areas (caused by melanins). Species in this group, which is also called neuropogonoid (Lumbsch and Wirtz 2011; Wirtz et al. 2008), can be difficult to distinguish by their general appearance and hence, molecular data, such as DNA marker sequencing, can be helpful in delimiting lineages (Articus 2004; Lumbsch and Wirtz 2011; Seymour et al. 2007; Wirtz et al. 2008; Wirtz et al. 2012). Earlier studies based on morphological and chemical data considered the neuropogonoid species as a subgenus Neuropogon in *Usnea* (Lamb 1964; Walker 1985) or as a distinct genus *Neuropogon* (Krog 1976; 1982; Lamb 1939). Molecular studies confirmed *Usnea* (including *Neuropogon*) as a monophyletic genus within Parmeliaceae (Crespo et al. 2007); however, the relationship of *Neuropogon* and *Usnea* remained ambiguous. A two-marker DNA analysis of *Usnea* elevated *Neuropogon* to a generic rank (Articus 2004), but subsequent studies provided evidence that *Neuropogon* is polyphyletic with a core group nested within *Usnea* (Seymour et al. 2007; Wirtz et al. 2008; Wirtz et al. 2012; Wirtz et al. 2006). Multi-locus DNA sequence data could not delimit individuals of the species *U.antarctica* and *U.aurantiacoatra* (Seymour et al. 2007; Wirtz et al. 2012) suggesting that they might be conspecific. In contrast, a recent microsatellite study provided evidence that the two species represent isolated lineages (Lagostina et al. 2018). Given the contradicting results of multi-locus and microsatellite data, we decided to employ a reduced genomic dataset to revisit the species delimitation of *U.antarctica* and *U.aurantiacoatra*.

The advent of next-generation sequencing (NGS), also referred to as high-throughput sequencing, drastically changed the scale of molecular datasets for systematic analyses and revolutionised our ability to assess evolutionary histories of organisms (Kraus and Wink 2015; Wachi et al. 2018; Zimmer and Wen 2015). Many molecular studies, such as the former species delimitation efforts for neuropogonoid *Usnea* spp., were limited to, at most, a dozen markers because their production would require tedious lab work and costly Sanger-sequencing (Hoffman and Lendemer 2018; Wilkinson et al. 2017). Population genomics of closely related organisms often relied on the descriptive power of microsatellite markers (Hodel et al. 2016). Compared to these traditional lab methods, NGS techniques allow a relatively straight-forward production of genome-scale datasets. Direct sequencing NGS methods, such as de-novo genome sequencing (Ellegren 2014), re-sequencing (Stratton 2008) or RNAseq of expressed genes (Ozsolak and Milos 2011; Wickett et al. 2014), can provide whole genome-scale data but may still be limited by high sequencing costs. Therefore, these methods are rarely applied to population studies which require the sequencing of many individuals. However, often a subset of the genome entails sufficient polymorphisms to answer questions of phylogenetic or population genomic studies. Hence, many NGS methods for systematic analyses are designed to be economical and generate reduced genome representation datasets (Allendorf 2017; Davey et al. 2011). One of these reduced representation methods is genotype-by-sequencing and its altered approach, which is known as restriction associated DNA sequencing (RADseq) (Baird et al. 2008). We recently designed a RADseq approach for metagenomic data derived from symbiotic lichen genomes, which allows reduced representation genomic analyses of numerous individuals for population-scale studies (Grewe et al. 2017).

By using genome-wide single nucleotide polymorphisms (SNPs) produced by restriction site-associated DNA sequencing (RADseq) of predominantly Antarctic lichen-forming fungi, our main aim in this study was to clarify the taxonomy of asexual *Usneaantarctica* and sexual *Usneaaurantiacoatra* and test their hypothesised species pair relationship. To further support our findings, we applied population genomic methods to measure the degree of genomic divergence and infer the levels of co-ancestry for each species.

## Methods

### Sample collection and site description

Samples were collected in Antarctica between December 2015 and January 2017. From these collected specimens, we chose to compare 105 representative specimens of the species *U.antarctica* and *U.aurantiacoatra* for this study (see details of specimens in Suppl. material 1). All selected specimens were either collected on King George Island (65) and Elephant Island (19) of the South Shetland Islands or in the Northern part of the Antarctic Peninsula (21) near the research bases Esperanza and Primavera. Fifty-eight of the 105 selected specimens were identified as *U.antarctica* and 47 specimens were identified as *U.aurantiacoatra* based on their phenotypical characters (Walker 1985). As a reference sequence to filter for lichen-fungal loci of *U.antarctica* and *U.aurantiacoatra* during the RADseq processing, we sequenced a specimen of *U.strigosa* that was collected in Arkansas, U.S.A. (Suppl. material 1).

### DNA extraction

Total metagenomic DNA was extracted either by following a cetyltrimethylammonium bromide (CTAB) protocol as modified by Cubero et al. (1999) or by using the ZR Fungal/Bacterial DNA MiniPrep Kit (Zymo Research, Irvine, CA, USA) as recommended by the manufacturer. We used the whole lichen thalli for DNA extraction from *U.antarctica* and *U.aurantiacoatra*, but only the central axis in *U.strigosa* to preferentially extract DNA from the lichen fungus (to avoid the photobiont). DNA concentrations of all samples were quantified with a Qubit fluorometer (Thermo Fisher Scientific, Waltham, MA, USA).

### Reference Sequencing and Assembly

We first deep-sequenced and assembled a reference sequence of an *Usneastrigosa* specimen to aid in mapping lichen-fungal loci during the processing of metagenomic RADseq data. A paired-end Illumina sequencing library of 150 bp read length was constructed from total DNA with the Nextera XT DNA Library Prep Kit (Illumina, San Diego, CA, USA) and sequenced on a NextSeq platform at the University of Illinois Chicago’s Sequencing Core Facility (Chicago, IL, USA). The resulting reads were quality trimmed using the programme Trimmomatic v0.36 (Bolger et al. 2014). Bases were trimmed when the average quality of 4-base sliding windows was below 15 and bases at the start and end of reads had a quality below 10. Subsequently, all trimmed reads, shorter than 25 bp, were filtered out (LEADING:10 TRAILING:10 SLIDINGWINDOW:4:15 MINLEN:25). The trimmed reads were used for a genome assembly with the programme SPAdes v3.5 (Bankevich et al. 2012) with default parameter settings. The assembled metagenomic scaffolds were loaded into the programme MetaWatt v3.5.3 (Strous et al. 2012) for a binning based on tetranucleotide frequencies. Scaffolds of fungal origin that clustered together were separated from the remaining scaffolds. All selected scaffolds that were larger than 10 kb were then included into the final reference sequence of *U.strigosa*. We used the Core Eukaryotic Gene Mapping Approach (CEGMA) to estimate the genomic completeness of the assembly (Parra et al. 2009). Finally, we created a Bowtie2 (Langmead and Salzberg 2012) database from the selected scaffolds for the mapping approach to filter for fungal RAD loci.

### RADseq Library Preparation and Sequencing

RADseq libraries for *Usneaantarctica* and *U.aurantiacoatra* were prepared as described previously (Grewe et al. 2017). In short, for the RADseq library production, DNA isolations were pooled with sequence adapters (Rubin and Moreau 2016), subsequently digested with the restriction enzyme ApeKI (New England Biolabs, Ipswich, MA, USA) and ligated using T4 ligase (New England Biolabs). Up to 48 samples with compatible barcodes were pooled and selected for fragments of sizes between 300 and 500 bp using the BluePippin DNA size selection system (Sage Science, Beverly, MA, USA). The pooled libraries were amplified using the REDTaq ReadyMix (Sigma-Aldrich, St. Louis, MO, USA) prior to sequencing on an Illumina MiSeq using the MiSeq Reagent Kit v3 for 150 cycles (Illumina, San Diego, CA, USA) to produce single-end sequences with a length of 150 bp.

### Assembly of RADseq datasets

The raw reads of *U.antarctica* and *U.aurantiacoatra* from the MiSeq sequencing were processed and assembled as described earlier for metagenomic datasets of lichens (Grewe et al. 2017). This process used a combination of the ipyRAD (https://github.com/dereneaton/ipyrad/blob/master/docs/index.rst) and pyRAD (Eaton and Ree 2013) pipelines with an additional mapping step that filtered for lichen-fungal loci with a reference sequence. Subsequently, we refer to the raw Illumina RAD sequences as ‘read’ and name the clustered reads per individual sample ‘loci’; the final matrices are alignments of homologous loci from multiple samples with nucleotide substitutions referred to as ‘SNP’. In pyRAD, we set the datatype to genotype-by-sequencing (gbs), ploidy to haploid (1), a similarity threshold for the clustering of reads within and between individuals to 90% (.90) and a minimum coverage of four individuals per locus (4). For the reference-based filtering of RAD loci, we used Bowtie2 with adjusted parameters to allow one permitted mismatch (−N 1), a seed length of 20 (−L 20), up to 20 seed extension attempts (−D 20) and a maximum “re-seeding” of 3 (−R 3). Following an initial round with all sequenced samples, we re-ran step 7 of pyRAD and excluded samples with less than 1000 recovered loci. We used the filtered pyRAD output files, such as unlinked_snps, alleles and vcf, for further analyses.

### Phylogenetic reconstructions

Phylogenetic trees were calculated from all unlinked SNPs of the filtered RADseq dataset, i.e. a matrix that was limited to one SNP per RAD locus. This matrix was used for a RAxML v7.2.8 (Stamatakis 2006) maximum likelihood analysis using the GTR + G model. For each analysis, 100 bootstrap replicates were calculated using the fast bootstrapping option implemented in RAxML (Stamatakis et al. 2008). The resulting phylogenetic tree was midpoint rooted and drawn to scale with FigTree v1.4.3 (http://tree.bio.ed.ac.uk/software/figtree/).

### Analysis of population structure

To calculate differences in the population structure between *U.antarctica* and *U.aurantiacoatra*, we created a reduced dataset that included all sites with a minor allele frequency (MAF) greater than or equal to 0.05 and greater than 50% coverage using vcftools v0.1.15 (Danecek et al. 2011). This reduced vcf file was converted into a genind object from the R package adegenet v2.0.2 (Jombart and Ahmed 2011; Jombart et al. 2010). The genind object was appended with additional information settings for haploid genomes and the population memberships for samples according to their initial identification based on morphological characters. With all information enclosed, the genind object became subject to population genetics analyses encoded in R.

To determine the degree to which both populations are subdivided, we estimated Gst (Nei 1973) and Hedrick’s standardised genetic differentiation measures G'st (Hedrick 2005) and Jost’s D (Jost 2008) by using the R package mmod v1.3.3 (Winter 2012). Gst is a good measure when the mutation rate is small relative to migration rate; contrarily, G’st and D fit to data with high mutation rates and two populations (Whitlock 2011). We used these multiple statistics to get a comprehensive measure of genomic differentiation. In a population pairwise comparison, we calculated these indices per site and plotted the values by frequency in separate histograms for Gst, G’st and D.

The genetic structure of samples of *U.antarctica* and *U.aurantiacoatra* was evaluated with the Discriminant Analysis of Principal Components (DAPC) implemented in the R package adegenet v2.02. This non-parametric method first transforms the data using a principle components analysis (PCA) and subsequently distinguishes between two or more groups using a discriminant analysis (DA). The DAPC was conducted by using the first 60 principal components and all (two) DA-eigenvalues. In addition to the display of the genetic variation in genomic space, the DAPC allows a prediction of the group membership probability for each sample which is visualised in a STRUCTURE-like plot.

In addition to the nonparametric approach with DAPC, we used a model-based method to detect population subdivision using the programme fineRADstructure (Malinsky et al. 2018). This software is specifically designed to measure population structure amongst haplotypes inferred from RADseq datasets. We used the script finerad_input.py included in fineRADstructure-tools (https://github.com/edgardomortiz/fineRADstructure-tools) to convert the pyRAD alleles output into the input format for fineRADstructure. During the conversion, we also reduced the dataset to only unlinked loci (default parameter) with a minimum sample number of 4 (--minsample 4). As recommended by the authors, we then re-ordered the unsorted RAD loci with the script sampleLD.R which is part of the fineRADstructure package. Next, we used the scripts, RADpainter and fineSTRUCTURE, which are both implemented in fineRADstructure, to measure the population structure. First, we calculated the co-ancestry matrix with RADpainter for haploid datasets (-p 1). We then used fineSTRUCTURE for the Markov chain Monte Carlo (MCMC) clustering algorithm with the following arguments: -x 100,000, -z 100,000 and -y 1,000. We also started fineSTRUCTURE with the arguments -m T and -x 10,000 to run a simple tree-building algorithm on the data of the co-ancestry matrix. Finally, the co-ancestry matrix, MCMC output and the coalescence tree were loaded into the programme ‘Finestructure GUI’ for visualisation.

### Reproducibility

The *U.strigosa* reference sequence and all scripts that were used in this study are available online (https://github.com/felixgrewe/Usnea). All RAD sequences were deposited in the NCBI Sequence Read Archive (SRA) with accession number PRJNA505526.

## Results

### Reference assembly and RADseq results

We assembled a draft reference genome of *U.strigosa* to filter for fungal RAD loci from *U.antarctica* and *U.aurantiacoatra*. The Illumina NextSeq sequencing of the whole *U.strigosa* lichen resulted in 8,552,530 metagenomic paired-end reads. First, we trimmed these raw data which reduced the paired-end reads to 8,366,962 (97.78% of raw data). The trimmed read pairs were then assembled into 16,932 scaffolds (N50 = 12,750 bp) with a total size of 40.9 Mbp (including 1,187 scaffolds of sizes larger than 10 kb). Metagenomic binning identified 28.92 Mbp of the assembly as fungal derived from which we selected 1,100 scaffolds (N50 = 23,562 bp) with sizes larger than 10 kb; all but two of these scaffolds were continuous assemblies (contigs). The sorted draft genome of *U.strigosa* had a total size of 24.1 Mbp and an estimated completeness of 72.18%.

We included 105 specimens of *U.antarctica* and *U.aurantiacoatra* that were collected in Antarctica in four RADseq libraries (Suppl. material 2). The sequence read number of each sample varied widely from 13,659 for sample EL0059 to 1,942,819 for sample EL0074 with an average sequence read number of 488,468 (sd = 313,604). The number of loci (within sample clusters) that pyRAD generated from these sequences directly correlated with the initial number of sequences (R2 = 0.8017, Suppl. material 3). An average of 21.8% (sd = 2.9%) of all loci mapped to the lichen fungus reference genome and, of these, an average of 85.4% (sd = 5.5%) were included into the final pyRAD dataset. The numbers of loci before and after the mapping were directly correlated (R2 = 0.7598, Suppl. material 3); however, the number of mapped loci reached saturation at an average of 6,496 (sd = 801) for samples with more than 40,000 initial loci. In addition, the number of mapped loci were strongly correlated to the number of loci included in the final dataset (R2 = 0.9869, Suppl. material 3). Two samples of *U.antarctica* (EL0059, EL0281) and two samples of *U.aurantiacoatra* (EL0415, EL0437) had less than 1,000 loci in the final dataset and were removed from the analysis. All remaining 101 samples in the final dataset had on average of 4,143 (sd = 1,316) loci (Suppl. material 2).

### Phylogenetic analysis of RADseq data

**Figure 1.**
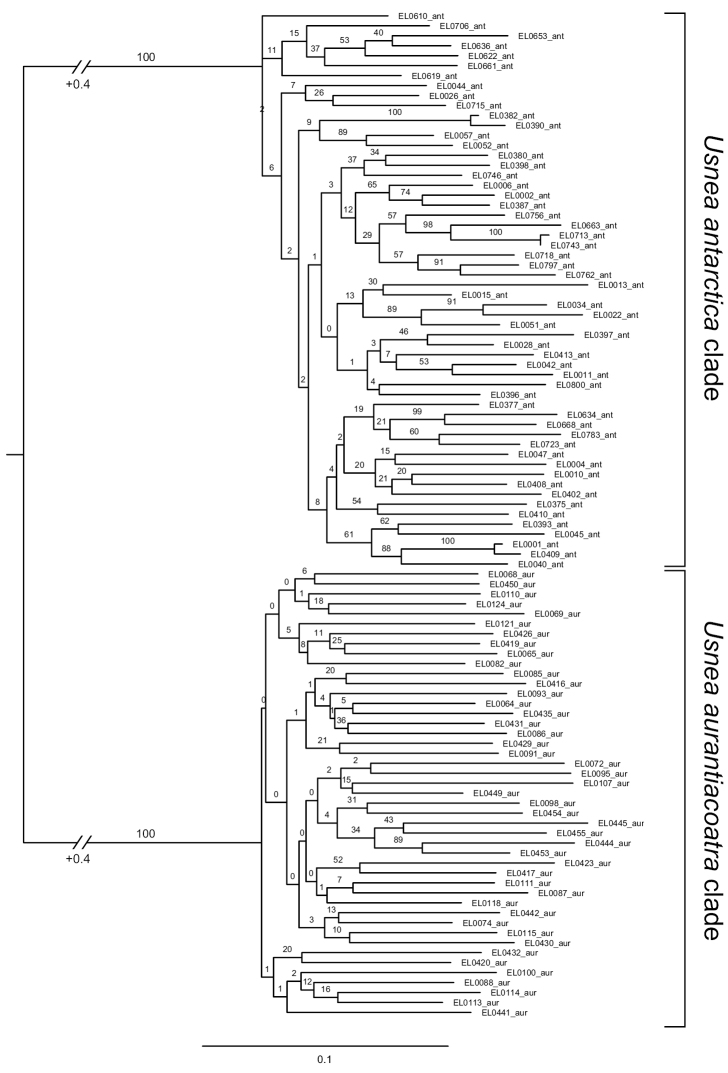
Phylogenetic tree inferred from the *U.antarctica* and *U.aurantiacoatra*RADseq data. The clades of each species are highlighted by brackets. Bootstrap values are indicated at the branches. The unit of branch length is substitutions per site. Note that branches leading to both major clades were abbreviated by 0.4 substitutions per site.

The phylogenetic analysis of the RADseq data showed two distinct and highly supported clades corresponding to the phenotypically circumscribed species *U.antarctica* and *U.aurantiacoatra* (Figure 1). The phylogenetic tree was calculated from a matrix with 7,087 positions and 53.24% gaps. Most internal relationships within each clade remained unresolved; however, the *U.antarctica* clade showed higher internal support values than the *U.aurantiacoatra* clade. Within the *U.antarctica* clade, three sister relationships of *U.antarctica* (EL0001 and EL0409, EL0382 and EL0390, EL0713 and EL0743) had a 100% bootstrap support and short branches, indicating low genomic divergence.

### Population genomic analyses of RADseq data

**Figure 2.**
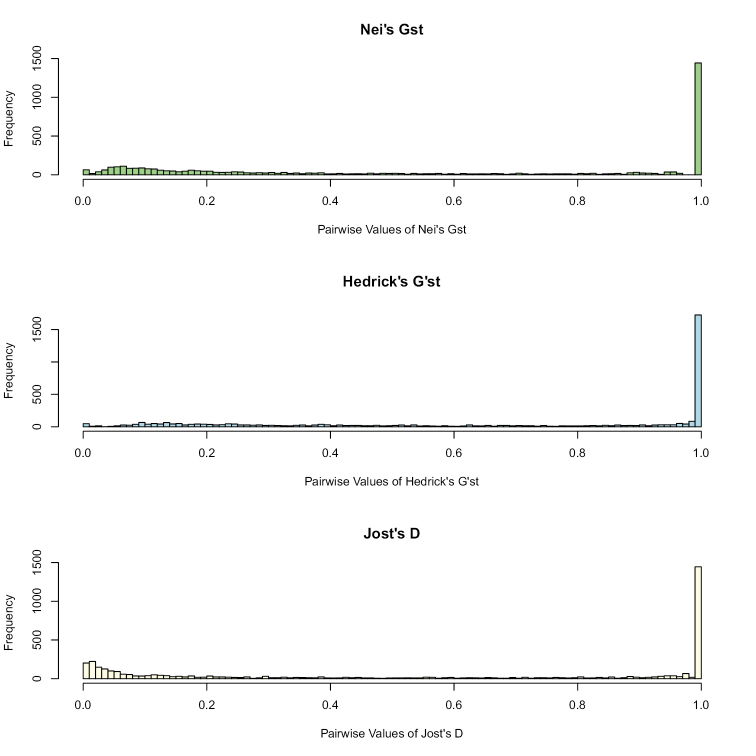
Pairwise Gst, G’st and D distribution. Pairwise values of Nei’s Gst (green), Hedrick’s G’st (blue) and Jost’s D (yellow) are plotted by their frequency.

We determined the degree to which both species complexes are subdivided by Gst, G’st and D measurements. For these analyses, we included only SNPs with a MAF greater than 0.05 and more than 50% coverage. This reduced the RAD dataset to a total of 4,132 SNPs. We plotted the frequency of Gst, G’st and D measures for each SNP (Figure 2). A strong tendency towards 1 for most SNPs in all three measures strongly indicated that genomes of both species were completely isolated. This was also supported by the average measures of Gst, G’st and D of 0.70, 0.93 and 0.60, respectively.

**Figure 3.**
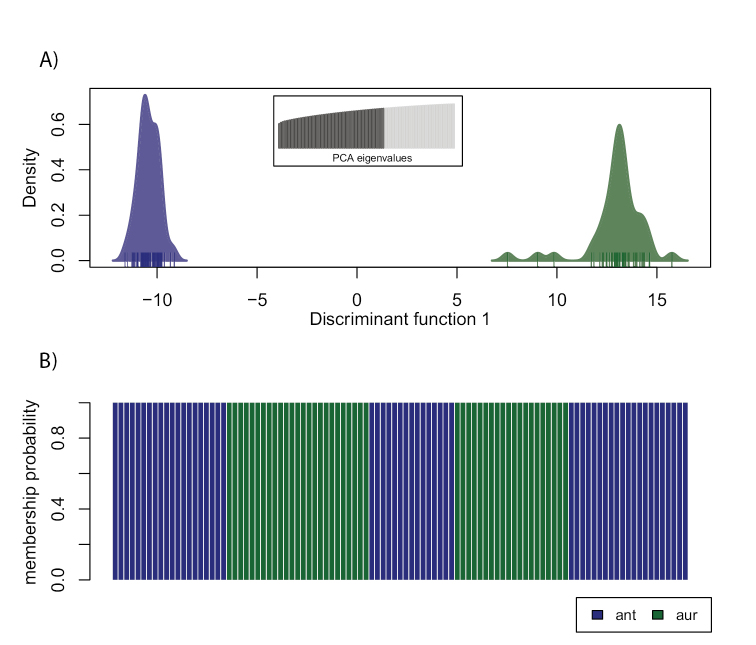
Genomic variation by non-parametric DAPC. **A**DAPC plot of the densities of *U.antarctica* (blue) and *U.aurantiacoatra* (green) on the first retained discriminant function **B** Bar plot of group membership probabilities.

The same reduced dataset of 4,132 SNPs was used to differentiate the genomes by their variation in a non-parametric approach with a DAPC (Figure 3A). The DAPC combines a PCA with a DA for a separation of genomes based on their variance between groups rather than the total variance of the sample. The resulting clusters of both species were clearly separated in genomic space and showed no evidence for admixture. In addition, the group membership probabilities indicated absolute discrimination of the two species by the DAPC assigning each individual with 100% probability to their respective species (Figure 3B).

**Figure 4.**
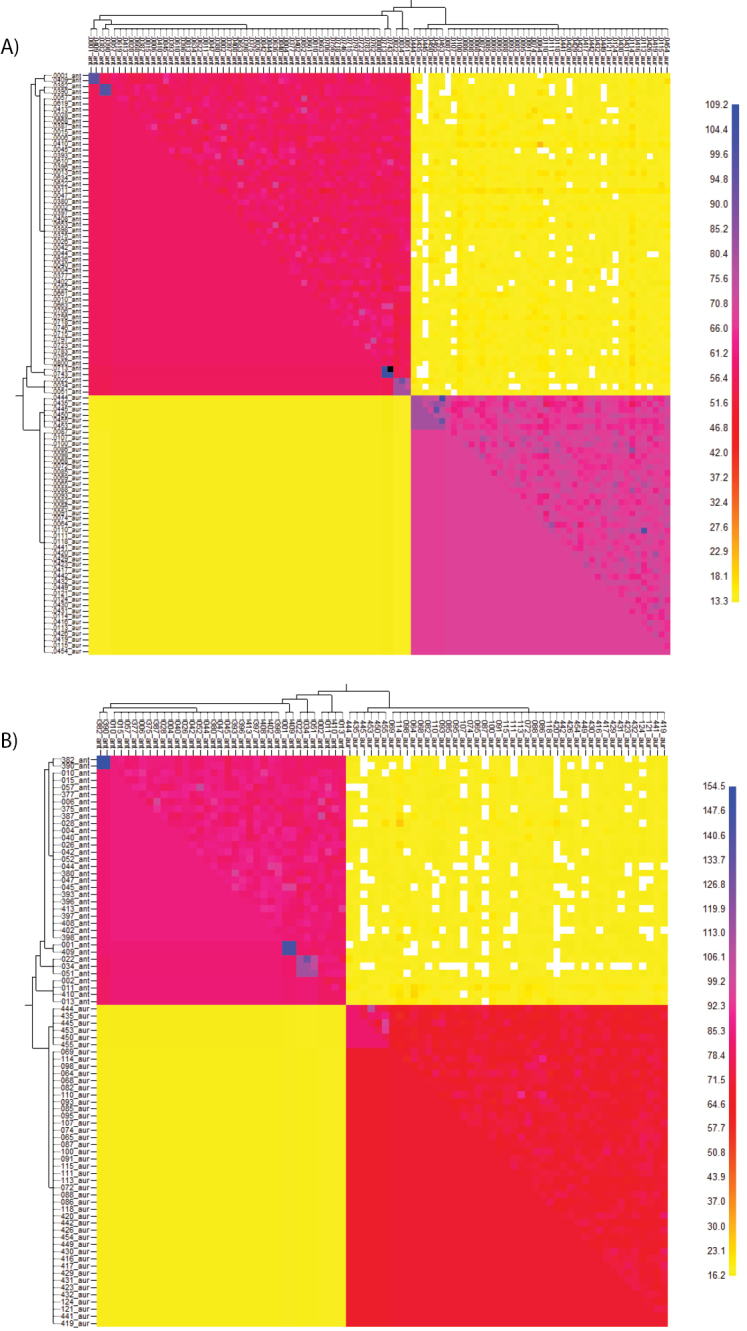
Clustered fineRADstructure co-ancestry matrix. **A** Full dataset including *U.antarctica* collected on the Antarctic Peninsula in addition to *U.antarctica* and *U.aurantiacoatra* collected on King George Island and Elephant Island **B** Reduced dataset with all *U.antarctica* and *U.aurantiacoatra* collected on King George Island and Elephant Island. Two major clades are corresponding to the two species *U.antarctica* (top-left) and *U.aurantiacoatra* (bottom-right). The top and left trees were calculated from the co-ancestry matrix to sort the individuals by their population structure. The matrix is diagonally split into the top-right half showing raw data and the bottom-left half displaying aggregated data.

The separation of *U.antarctica* and *U.aurantiacoatra* was further supported by the results of a Bayesian model-based approach with the programme fineRADstructure. By converting the pyRAD allele output for fineRADstructure, we reduced the dataset to 3,803 unlinked SNPs with a minimum coverage of 4 samples. The resulting clustered co-ancestry matrix showed that both species shared more co-ancestry within each other than between species (Figure 4). By comparing both species clusters, *U.aurantiacoatra* showed a higher estimated co-ancestry than *U.antarctica* (Figure 4A). To avoid a sampling bias, we reduced the dataset for the fineRADstructure analysis to include only samples collected on King George Island and Elephant Island. This reduced the dataset to 80 samples and 3,652 unlinked SNPs with a minimum coverage of 4 samples. The resulting plot of the reduced dataset also showed higher shared co-ancestry within each species compared to that between species, but estimated higher co-ancestry of *U.antarctica* than *U.aurantiacoatra* (Figure 4B), opposite to the entire dataset. In addition, both matrices visualised different degrees of intraspecific co-ancestry and suggested substructure for a group of three specimens of *U.antarctica* from Potter Peninsula, King George Island (EL0022, EL0034 and EL0051) and for six specimens of *U.aurantiacoatra* from Fildes Peninsula, King George Island (EL0444, EL0435, EL0445, EL450, EL0455 and EL0453). Moreover, two *U.antarctica* specimen pairs collected on King George Island (EL0001 and EL0409, EL0382 and EL390) and one pair collected on the Antarctic Peninsula (EL0713 and EL0743) showed the highest degrees of co-ancestry demonstrating very close relatedness, such as sister or clonal relationships. These results agreed with the phylogenetic inference (see above) in which the same *U.antarctica* specimens were close sister taxa.

## Discussion

In this study, we used RAD sequencing for evaluating the delimitation of two predominantly Antarctic *Usnea* species. Phylogenetic evidence and population genomic analyses of the RADseq data strongly supported that the two species represent independent lineages. Although both species showed no overlapping genomic structure in a DAPC, we could compare levels of co-ancestry and detect genomic substructure within each species in a fineRADstructure plot.

In previous studies using multi-locus approaches, including the ITS barcoding marker (Schoch et al. 2012), the relationship of *U.antarctica* and *U.aurantiacoatra* remained unresolved and, since specimens of both species did not separate as different clades, conspecificity of the species was not ruled out (Seymour et al. 2007; Wirtz et al. 2012). Our study using RADseq supports the results obtained using microsatellite data that suggested the two species are distinct lineages (Lagostina et al. 2018). In *U.antarctica* and *U.aurantiacoatra*, the taxonomic interpretation of species pairs as separate species (Poelt 1972) is supported.

We developed a RADseq method for lineages involved in intimate symbiotic associations (Grewe et al. 2017), which we here successfully implemented for the use of delimiting two species. Different to the previously described RADseq method that used a reference genome from a lichen-fungal culture, we successfully generated a reference genome from a metagenomic *de-novo* assembly of *U.strigosa*. The filtering of the metagenomic assembly for fungal derived content reduced the size and completeness of the fungal reference (28.92 Mbp, CEGMA: 72.18%) compared to the reference genome assembly from a lichen-fungal culture which was used in earlier studies (31.6 Mbp, CEGMA: 96.77%) (Grewe et al. 2017; Leavitt et al. 2016b). However, the saturation of successfully mapped loci to the reference (Suppl. material 3) suggested that the maximum number of possible mapped loci was reached for samples with many initial loci. Therefore, although using a smaller reference and less fungal derived loci than in our initial study (Grewe et al. 2017), this RADseq approach still was successful in mapping a large number of fungal loci sufficient for phylogenetic and population genomic methods. This widens the potential application of RADseq for intimate symbiotic organisms and includes studies where cultures of one symbiotic partner are not readily available.

RADseq data are extremely powerful, since the method generates a matrix of thousands of homologous loci derived from randomly distributed regions across the genome. Many studies have successfully used large RADseq datasets for phylogenetic analysis which were difficult to resolve due to insufficient signals in available markers (Eaton and Ree 2013; Escudero et al. 2014; Hipp et al. 2014; Vargas et al. 2017; Wagner et al. 2018). Our phylogenetic and population genomic results from the RADseq dataset clearly delimited *U.antarctica* and *U.aurantiacoatra* into two lineages (Figures 1–4) supporting the acceptance of two species. This confirms that closely related species are difficult to separate using sequence-based multi-locus approaches and great care should be taken when interpreting results from molecular studies when it comes to testing for conspecificity. On the other hand, the microsatellite-based multi-locus approach by Lagostina et al. (2018) rendered almost identical results, including nearly 100 % correct assignment of samples to their species.

The fineRADstructure matrix estimated lower co-ancestry (and hence higher genotypic variation) for the sexually-reproducing *U.aurantiacoatra*, compared to the asexually-reproducing *U.antarctica* when comparing samples that were collected in the same geographic range (Figure 4B). This result agrees with earlier observations that asexual populations have lower genotypic variation than sexual populations in modelling approaches (Balloux et al. 2003) and empirical measures (Delmotte et al. 2002). Moreover, Lagostina et al. (2018) inferred lower genetic variability for *U.antarctica* than *U.aurantiacoatra* using 23 microsatellite loci. These authors also used samples collected in mixed stands of both species from King George and Elephant Island. When we increased our sampling of *U.antarctica* to include a much wider geographical range (Antarctic Peninsula in addition to King George and Elephant Island) compared to the sampling of *U.aurantiacoatra* (King George and Elephant Island only), the matrix indicated increased levels of co-ancestry and a lower genotypic variation (Figure 4A). Although this comparative analysis is lacking collections of *U.aurantiacoatra* from the Antarctic Peninsula for a direct comparison, it should be noted that *U.antarctica* covers a wider geographical range than *U.aurantiacoatra* (Walker 1985) and this wider species distribution might increase genetic variability. The difference in distribution may result from the main form of reproductive units of both *Usnea*. The exclusively sexual *U.aurantiacoatra* reproduces via the dispersal of fungal spores which are required to meet with an appropriate photobiont after germination. The asexual *U.antarctica* on the other hand is in majority vegetatively reproducing via soredia, which already include the photobiont. Therefore, even if both reproductive units are dispersed over similar distances, the success rate of colonisation may be higher for soredia and explain the overall wider distribution and therefore genetic variability of *U.antarctica*. Finally, it was predicted that a small number of sexual individuals per generation — and *U.antarctica* rarely can be found with apothecia — is sufficient to make an apparently asexual population highly variable (Bengtsson 2003).

Despite the lower co-ancestry of *U.antarctica* compared to *U.aurantiacoatra*, we detected three pairs of very close relatives with high co-ancestry of *U.antarctica* (Figure 4). The three pairs were collected on Elephant Island, King George Island and on the Antarctic Peninsula, respectively and may indicate almost immediately related clones. On Elephant Island and the Antarctic Peninsula, the pairs were collected in the same locations with a greater chance to pick up clones. However, the clonal pair from King George Island must have dispersed between Fildes and Potter Peninsula over ice or water boundaries prior to our collection. Contrarily, none of the individuals of *U.aurantiacoatra* expressed similarly close relationships. However, we could detect substructure for a group of six individuals of *U.aurantiacoatra* collected at the same location and three specimens of *U.antarctica* collected at different locations on King George Island, which indicates the potential of this analysis to identify (sub)population structure. Using this detailed method to measure co-ancestry on a deeper sampling of individuals of *Usnea* may, in future, provide a comprehensive picture of population structure and diversification.

## Conclusion

We successfully used RADseq for phylogenetic and population genomic studies on two species of the lichen-fungal genus *Usnea*. Phylogenetic inference using RAD data clearly delimited the species *U.antarctica* and *U.aurantiacoatra* into two lineages, which were irresolvable using multi-locus DNA sequence markers. Furthermore, the RADseq approach offered sufficient genotyping data for conclusive population genomic analyses. We used RADseq to measure lower co-ancestry in the asexual *U.antarctica* than in the sexual *U.aurantiacoatra*, potentially derived from a wider geographical distribution of *U.antarctica* in our sample. These results show that RADseq has much potential for future phylogenetic and population genomic studies on lichens, particularly for groups of organisms which remained unresolved by multi-locus markers.

## Supplementary materials

Supplementary material 1 Origin of samples used for this study Data type: species data This is an open access article distributed under the terms of the Creative Commons Attribution License (CC BY 4.0), which permits unrestricted use, distribution, and reproduction in any medium, provided the original author and source are credited.Felix Grewe, Elisa Lagostina, Huini Wu, Christian Printzen, H. Thorsten Lumbsch

Supplementary material 2 Overview of RADseq results after individual steps of RAD analyses Data type: molecular data This is an open access article distributed under the terms of the Creative Commons Attribution License (CC BY 4.0), which permits unrestricted use, distribution, and reproduction in any medium, provided the original author and source are credited.Felix Grewe, Elisa Lagostina, Huini Wu, Christian Printzen, H. Thorsten Lumbsch

Supplementary material 3 Correlation of RADseq results after individual steps of RAD analyses Data type: molecular data This is an open access article distributed under the terms of the Creative Commons Attribution License (CC BY 4.0), which permits unrestricted use, distribution, and reproduction in any medium, provided the original author and source are credited.Felix Grewe, Elisa Lagostina, Huini Wu, Christian Printzen, H. Thorsten Lumbsch
