# Supplementary material for: Population genomic analyses of RAD sequences resolves the phylogenetic relationship of the lichen-forming fungal species Usneaantarctica and Usneaaurantiacoatra
Source: MycoKeys. 2018 Dec 12;(43):91–113. doi: 10.3897/mycokeys.43.29093 (PMC6300515; doi:10.3897/mycokeys.43.29093)
Supplement: Supplementary material 1 — Origin of samples used for this study [file mycokeys-43-091-s003.pdf]

Specimen Number	Species Name	MiSeq sequencing	Within sample cluster (loci)	Loci mapped to refernce	Percentage mapped	Loci in final dataset	Loci in final dataset (>1000)
EL0001	Usnea antartica	556,958	22,561	4,915	21.8%	4,106	4,108
EL0002	Usnea antartica	156,335	11,131	2,559	23.0%	2,393	2,393
EL0004	Usnea antartica	428,463	20,713	4,934	23.8%	4,081	4,083
EL0006	Usnea antartica	294,306	19,364	4,424	22.8%	4,133	4,133
EL0010	Usnea antartica	347,330	16,384	3,718	22.7%	3,006	3,008
EL0011	Usnea antartica	307,277	16,342	3,681	22.5%	3,029	3,026
EL0013	Usnea antartica	376,494	19,259	4,570	23.7%	3,772	3,774
EL0015	Usnea antartica	406,654	22,695	5,103	22.5%	4,665	4,666
EL0022	Usnea antartica	210,615	12,658	3,029	23.9%	2,380	2,382
EL0026	Usnea antartica	1,639,547	36,253	8,246	22.7%	7,169	7,170
EL0028	Usnea antartica	201,408	13,476	3,070	22.8%	2,852	2,852
EL0034	Usnea antartica	188,669	13,088	3,006	23.0%	2,799	2,799
EL0040	Usnea antartica	532,132	21,068	4,405	20.9%	3,671	3,673
EL0042	Usnea antartica	626,833	23,759	5,558	23.4%	4,720	4,722
EL0044	Usnea antartica	799,833	28,368	6,640	23.4%	5,750	5,752
EL0045	Usnea antartica	174,992	9,665	2,290	23.7%	1,733	1,735
EL0047	Usnea antartica	453,480	24,895	5,778	23.2%	5,210	5,211
EL0051	Usnea antartica	803,397	28,199	6,695	23.7%	5,798	5,800
EL0052	Usnea antartica	126,118	8,419	1,920	22.8%	1,777	1,777
EL0057	Usnea antartica	557,873	27,533	5,783	21.0%	5,200	5,202
EL0059	Usnea antartica	13,659	63	10	15.9%	10	
EL0064	Usnea aurantiaco-atra	116,561	7,525	1,747	23.2%	1,571	1,572
EL0065	Usnea aurantiaco-atra	202,688	12,670	2,898	22.9%	2,443	2,442
EL0068	Usnea aurantiaco-atra	1,023,871	47,973	5,786	12.1%	4,883	4,887
EL0069	Usnea aurantiaco-atra	638,013	26,113	6,105	23.4%	5,257	5,261
EL0072	Usnea aurantiaco-atra	329,200	17,358	4,181	24.1%	3,425	3,429
EL0074	Usnea aurantiaco-atra	1,942,819	55,459	7,531	13.6%	6,381	6,385
EL0082	Usnea aurantiaco-atra	313,415	16,611	3,839	23.1%	3,098	3,103
EL0085	Usnea aurantiaco-atra	589,806	29,379	4,360	14.8%	3,562	3,566
EL0086	Usnea aurantiaco-atra	643,322	25,389	6,099	24.0%	5,289	5,293
EL0087	Usnea aurantiaco-atra	538,540	22,856	4,833	21.1%	3,997	4,001
EL0088	Usnea aurantiaco-atra	1,002,560	30,095	7,087	23.5%	6,214	6,218
EL0091	Usnea aurantiaco-atra	578,617	26,430	5,721	21.6%	4,786	4,790
EL0093	Usnea aurantiaco-atra	711,192	29,078	5,441	18.7%	4,520	4,525
EL0095	Usnea aurantiaco-atra	383,863	18,990	4,589	24.2%	3,757	3,761
EL0098	Usnea aurantiaco-atra	304,105	15,797	3,633	23.0%	2,890	2,894
EL0100	Usnea aurantiaco-atra	167,610	9,691	2,228	23.0%	1,967	1,965
EL0107	Usnea aurantiaco-atra	244,936	14,771	3,617	24.5%	2,857	2,861
EL0110	Usnea aurantiaco-atra	88,501	5,732	1,379	24.1%	1,262	1,260
EL0111	Usnea aurantiaco-atra	755,220	31,945	5,190	16.2%	4,313	4,317
EL0113	Usnea aurantiaco-atra	837,008	36,040	5,563	15.4%	4,641	4,645
EL0114	Usnea aurantiaco-atra	128,508	8,310	1,917	23.1%	1,696	1,696
EL0115	Usnea aurantiaco-atra	401,037	19,826	4,814	24.3%	3,999	4,003

EL0118	Usnea aurantiaco-atra	623,414	29,417	5,001	17.0%	4,111	4,115
EL0121	Usnea aurantiaco-atra	1,256,791	34,172	8,018	23.5%	6,936	6,941
EL0124	Usnea aurantiaco-atra	287,888	15,019	3,635	24.2%	2,849	2,852
EL0375	Usnea antartica	608,102	24,878	5,924	23.8%	5,017	5,020
EL0377	Usnea antartica	457,290	25,467	5,385	21.1%	4,879	4,880
EL0380	Usnea antartica	650,500	34,318	6,348	18.5%	5,688	5,690
EL0381	Usnea antartica	57,322	1,624	312	19.2%	297	
EL0382	Usnea antartica	602,744	25,718	5,907	23.0%	4,994	4,996
EL0387	Usnea antartica	509,889	27,727	6,022	21.7%	5,389	5,390
EL0390	Usnea antartica	316,809	16,815	3,953	23.5%	3,178	3,180
EL0393	Usnea antartica	415,861	19,531	4,546	23.3%	3,787	3,789
EL0396	Usnea antartica	1,033,299	45,350	7,179	15.8%	6,280	6,282
EL0397	Usnea antartica	277,565	16,040	3,815	23.8%	3,069	3,071
EL0398	Usnea antartica	675,252	30,271	6,841	22.6%	5,994	5,996
EL0402	Usnea antartica	401,543	19,930	4,822	24.2%	3,948	3,950
EL0408	Usnea antartica	825,308	35,339	6,633	18.8%	5,881	5,883
EL0409	Usnea antartica	391,229	18,947	4,396	23.2%	3,582	3,584
EL0410	Usnea antartica	777,413	37,711	6,564	17.4%	5,819	5,820
EL0413	Usnea antartica	205,831	12,412	2,897	23.3%	2,265	2,267
EL0415	Usnea aurantiaco-atra	42,389	1,887	443	23.5%	408	
EL0416	Usnea aurantiaco-atra	751,019	25,530	5,761	22.6%	4,823	4,827
EL0417	Usnea aurantiaco-atra	623,045	26,611	6,309	23.7%	5,368	5,372
EL0419	Usnea aurantiaco-atra	1,288,183	49,256	5,905	12.0%	4,945	4,949
EL0420	Usnea aurantiaco-atra	622,806	31,966	5,148	16.1%	4,254	4,258
EL0423	Usnea aurantiaco-atra	453,929	18,105	4,071	22.5%	3,294	3,297
EL0426	Usnea aurantiaco-atra	348,399	18,815	4,644	24.7%	3,790	3,794
EL0429	Usnea aurantiaco-atra	573,360	22,980	5,163	22.5%	4,301	4,305
EL0430	Usnea aurantiaco-atra	344,277	18,128	4,423	24.4%	3,667	3,671
EL0431	Usnea aurantiaco-atra	735,439	27,213	5,587	20.5%	4,697	4,701
EL0432	Usnea aurantiaco-atra	656,918	26,007	6,354	24.4%	5,475	5,479
EL0435	Usnea aurantiaco-atra	174,671	7,725	1,880	24.3%	1,303	1,306
EL0437	Usnea aurantiaco-atra	77,221	3,090	746	24.1%	469	
EL0441	Usnea aurantiaco-atra	820,039	28,747	6,563	22.8%	5,699	5,703
EL0442	Usnea aurantiaco-atra	363,275	18,901	3,432	18.2%	2,607	2,612
EL0444	Usnea aurantiaco-atra	404,154	18,331	4,392	24.0%	3,551	3,554
EL0445	Usnea aurantiaco-atra	206,372	10,974	2,222	20.2%	1,583	1,586
EL0449	Usnea aurantiaco-atra	253,377	13,104	2,967	22.6%	2,211	2,214
EL0450	Usnea aurantiaco-atra	385,470	21,179	5,208	24.6%	4,356	4,360
EL0453	Usnea aurantiaco-atra	464,018	22,385	4,584	20.5%	3,643	3,647
EL0454	Usnea aurantiaco-atra	817,117	28,409	6,900	24.3%	5,901	5,905
EL0455	Usnea aurantiaco-atra	460,912	22,076	5,370	24.3%	4,488	4,492
EL0610	Usnea antartica	789,315	33,454	6,929	20.7%	6,087	6,089
EL0619	Usnea antartica	357,139	22,189	5,154	23.2%	4,607	4,608
EL0622	Usnea antartica	274,638	18,005	4,172	23.2%	3,734	3,735
EL0634	Usnea antartica	388,312	20,748	4,806	23.2%	4,274	4,275
EL0636	Usnea antartica	496,502	23,176	4,114	17.8%	3,708	3,708
EL0653	Usnea antartica	244,311	15,567	3,682	23.7%	3,324	3,325

EL0661	Usnea antartica	740,280	48,830	6,075	12.4%	5,333	5,333
EL0663	Usnea antartica	382,187	22,285	4,814	21.6%	4,253	4,254
EL0668	Usnea antartica	450,918	25,702	5,615	21.8%	4,913	4,913
EL0706	Usnea antartica	330,495	18,424	3,987	21.6%	3,607	3,607
EL0713	Usnea antartica	286,634	17,397	3,799	21.8%	3,465	3,466
EL0715	Usnea antartica	226,991	14,425	3,290	22.8%	3,054	3,054
EL0718	Usnea antartica	537,289	25,927	5,999	23.1%	5,361	5,362
EL0723	Usnea antartica	688,297	29,979	6,517	21.7%	5,802	5,804
EL0743	Usnea antartica	672,100	31,321	6,351	20.3%	5,621	5,623
EL0746	Usnea antartica	361,687	22,251	5,186	23.3%	4,663	4,665
EL0756	Usnea antartica	257,965	18,131	4,316	23.8%	3,936	3,937
EL0762	Usnea antartica	339,665	20,967	4,808	22.9%	4,344	4,345
EL0783	Usnea antartica	335,663	21,587	5,159	23.9%	4,623	4,623
EL0797	Usnea antartica	306,453	19,557	4,485	22.9%	4,002	4,001
EL0800	Usnea antartica	438,075	24,271	5,491	22.6%	4,837	4,836
